# Measuring inequities in transportation injuries in a Canadian commuter cohort: impacts of individual versus neighbourhood income

**DOI:** 10.1186/s40621-025-00615-8

**Published:** 2025-10-01

**Authors:** Ryann E. Yeo, Michael Branion-Calles, Linda Rothman, Meghan Winters, M. Anne Harris

**Affiliations:** 1 Division of Epidemiology, Dalla Lana School of Public Health, University of Toronto, Toronto, Canada; 2 Department of Emergency Medicine, Faculty of Medicine, University of British Columbia, Vancouver, Canada; 3 School of Occupational and Public Health, Faculty of Community Services, Toronto Metropolitan University, Toronto, Canada; 4 Faculty of Health Sciences, Simon Fraser University, Burnaby, Canada

**Keywords:** Transportation injury, Pedestrian, Motor vehicle collision, Cyclist, CanCHECs, Individual income, Area-level income

## Abstract

**Background:**

Low income has been associated with a higher risk of transportation-related injury however, previous studies have largely relied on area-level income, due to the limited availability of individual-level data.

**Methods:**

To examine the independent and combined roles of individual- and area-level income, this prospective cohort study followed ~ 6,557,000 Canadians from the Canadian Census Health and Environment Cohorts (2006, 2011, 2016), for pedestrian, bicycling, or motor vehicle hospitalizations. Income was measured (1) individually by the low-income cut-off and (2) at the area level using neighbourhood income quintiles. Poisson regression estimated the incidence rate ratios (IRR) and 95% confidence intervals (CI) for transportation-related hospitalizations.

**Results:**

After adjusting for covariates, low-income individuals had higher risks of hospitalizations for pedestrian (IRR = 1.93, 95%CI (1.62, 2.29)), bicycling (IRR = 1.16, 95%CI (1.01, 1.34)) and motor vehicle injuries (IRR = 1.18, 95%CI (1.06, 1.31)). When both individual and neighbourhood income were assessed together we estimated, that those who lived in the lowest income neighbourhoods (compared to the highest) had a higher risk of pedestrian (IRR = 1.80, 95%CI (1.51, 2.14)) and motor vehicle injury (IRR = 1.33, 95%CI (1.22, 1.42)) but lower risk of bicycling injury (IRR = 0.73, 95%CI (0.65, 0.81)).

**Conclusions:**

The interaction between individual and neighbourhood income revealed an increased injury risk for low-income individuals in all neighbourhoods, with large inequities in pedestrian and motor vehicle injury risk persisting even in the highest-income neighbourhoods. These findings demonstrate individual income independently contributes to transportation injury risk, underscoring the importance of considering both individual- and area-level income.

**Supplementary Information:**

The online version contains supplementary material available at 10.1186/s40621-025-00615-8.

## Background

Income is a significant predictor of health disparities, and those of lower income have a greater risk of poorer health outcomes, including injury and death [1, 2]. In research on transportation injuries (those occurring while walking, cycling, or in a motor vehicle), income information has mainly been analyzed at the area level, due to the lack of data on income or other individual characteristics in injury records [3]. When area-level income is used, it is often used as a proxy for individual-level income, however, individual- and area-level income measures have been shown previously to have poor agreement [4]. 

When analyzed separately, individual- and area-level indicators of income can exhibit different associations with injury. Some studies have shown that individual income measures tend to yield stronger inequity estimates than area-level [5]. Additionally, when both are included, the interaction between the two indicators may also provide insight, for example, the different health risks between people who are low-income and reside in a low-income area compared to those who are low-income but reside in a high-income area [6]. 

Area-level ecologically assessed indicators of income (e.g., neighbourhood income) are commonly assumed in the literature to provide information on the collision environment. Pedestrian, cyclist and motor vehicle collisions occur more frequently in lower-income neighbourhoods, which may be due to a lack of safe road infrastructure in these areas [7]. These neighbourhoods tend to have fewer safe bike lanes [8], a higher volume of vehicles [7], and fewer speed humps [9]. 

Individual-level income has been investigated less frequently and not in relation to active transportation injuries (i.e. cyclists and pedestrians). However, there are income-related individual-level factors which have the potential to impact the risk of transportation injury. Low-income individuals experience poorer health outcomes in general, including comorbidities, which may increase risk and severity of transportation injuries [2, 10]. Other factors such as increased stress [11, 12], and fatigue [13, 14], or access to less safe vehicle models [15], may increase the risk of a collision. Employment, including occupation type, time of day commuting to work, and time spent commuting, may also play a part in one’s risk [14, 16, 17]. 

This study aims to investigate how individual-level income affects the risk of transportation hospitalizations in Canadian adults and to explore how individual income, neighbourhood income, and the interaction of the two, compare in predicting the risk of pedestrian, bicycling, and motor vehicle hospitalizations.

## Methods

### Data source

This study used the Canadian Census Health and Environment Cohorts (CanCHECs), which are population-based, probabilistically linked datasets [18]. CanCHECs link respondents of the Canadian Census and National Household Survey (NHS) to their administrative health data and postal codes [18]. 

The long-form Canadian Census is a mandatory survey which samples 20% of the population every five years [18]. The Census was replaced for one cycle in 2011 by the NHS, a voluntary survey which sampled 30% of the population and had a 69% response rate [18]. A description of the potential non-response bias can be found elsewhere [19]. The Discharge Abstract Database (DAD) was used to collect hospitalization data from hospital discharges and day surgery interventions for all provinces and territories, excluding Québec [20]. The Canadian Vital Statistics Death Database (CVSD) was used to obtain the death dates of cohort participants from all provinces and territories, although death data was not available for the Yukon from 2017 onward [21]. Three cycles of the Postal Code Conversion File Plus (PCCF+) were also used to link area-level income data to participants based on their postal code [22–24]. 

### Creation of the analytic cohort

To be included in the cohort, participants had to be usual residents of Canada on the day of the Census. CanCHECs linkage excluded those in institutions such as penitentiaries and nursing homes, and in 2011 specifically, those in collective households [18]. Generally, those who are young, mobile, low-income, homeless or Indigenous are also less likely to be captured by the long-form Census [18]. The cohort includes respondents from the 2006, 2011 and 2016 Census cycles who were followed from Census day until first hospitalization of any transportation injury, death or the end of the follow-up period, which was 1,789 days based on the current length of the 2016 linkage.

We analyzed a commuter cohort to leverage information about exposure to modes of travel contained within the Census. Commuters in the CanCHECs are those represented in “labour force” questions: 15 and older in private households with employment (either during the week of the Census or, if not currently employed, in at least one job since January 1  of that year) [25]. Census respondents who did not commute were those who reported no commute mode (*n* = 6,293,000) and were therefore not eligible for inclusion in the commuter cohort. Commuters represented 59.2% of the Census population (who had a successful linkage to the PCCF+).

We excluded participants who contributed less than one full day of follow-up, who were missing neighbourhood income, who were missing individual income, or who were from Québec (*n* = 2,585,000). Participants from Québec were removed as their hospitalization records are not included in the DAD. The analysis was conducted at the Toronto Research Data Centre using R 1.3.1073.

### Individual and neighbourhood income

Our exposure of interest was income, which we measured both individually and at the neighbourhood level. Individuals were classified as low-income if they met the low-income-cut-off after-tax (LICO) [26]. LICO refers to an income threshold at which a family spends 63% or more of their income on necessities such as food, shelter, and clothing [26]. If they spent less than 63%, then they would be considered non-low-income. While LICO is measured at the household level, it is then applied to individuals within the household. We used LICO as it is a more nuanced measure of low-income status that takes into account other factors, including family size and cost of living [27]. Throughout the paper, we use the term “individual income” to refer to the person-level classification of low-income status using LICO, based on household income.

We measured neighbourhood income at the level of the dissemination area (DA), which is the smallest spatial unit of the Census, typically containing 400–700 people [28]. The participant’s postal code for the year of the Census was obtained from mobility tax files, and we used the PCCF+ to assign an income quintile based on the postal code. For the 2016 participants, their neighbourhood income quintile is based on the average after-tax income of the DA, in which 1 is the lowest average income and 5 is the highest [24]. For participants from the 2006 and 2011 cycles, the quintile is based on the average income of the DA before tax [22]. DAs with less than 250 people were suppressed and their income quintile was imputed based on neighbouring DAs [29]. It should be noted that a postal code may serve more than one DA, and therefore, when this occurs, the DAs are randomly assigned to one of the DAs in proportion to the distribution of the population [24]. We are assuming in this study that the residential postal code is a reflection of the collision environment, as previous literature has shown transportation-related injuries often occur close to home and injury location is not otherwise available in this data [30]. 

Individuals with missing LICO data were removed from the cohort and this included participants in the territories and on reserves, as the individual income measure was not applicable there [26]. Those missing neighbourhood income were also removed. This missing data may be the result of missing or incomplete postal code information, which can occur as a result of emigration or unfiled taxes, among other reasons [18]. 

### Transportation injury hospitalizations

Our outcomes of interest were hospitalizations as a result of a pedestrian, cyclist, or motor vehicle occupant/driver-related injury. The cause of injury was recorded in linked health databases using the International Statistical Classification of Diseases, 10th Revision (ICD-10) system [31]. The ICD-10 codes were adapted from Branion-Calles et al. [32] and specific to victims who were pedestrians, cyclists, or motor vehicle occupants/drivers. Specifically, injuries indicating ‘pedal cycle’ involvement (ICD-10 codes beginning “V1”) were included as cycling injuries, and those beginning V0 considered pedestrian injuries. Motor vehicle driver or occupant injuries were those using V400-V699 codes in which the victim was the driver, passenger, or was unspecified (Appendix A: Table 1A).

### Commute mode as an indication of transportation mode exposure

Canada lacks national data on household travel to adjust observed injury counts for exposure to risk (distance, time, or number of trips travelled by a specific mode) [33]. We adapted methods previously reported to use the mode of commute as an indicator of exposure to risk [32]. Briefly, participants reported the commute mode used for the job reported in the Census module (usually, the job held during the week of the Census). If participants used multiple modes during their commute, they are asked to report the mode used for the greatest distance. We categorized this into pedestrian commute, bicycling commute, motor vehicle (car, truck, or van) commute, and other commute. The “other” group included commute by motorcycle or public transit. We then used commute mode as a covariate in the injury risk models.

### Covariates

In addition to commute mode exposure, we adjusted for several possible covariates to enable adjusted comparisons of the impacts of individual-level and neighbourhood-level income on injury risk. Covariates included age group: (15–24, 25–34, 35–44, 45–54, 55–64, 65+), sex (male, female), racialization (white, visible minority, Indigenous), recent immigration (immigrated within past 5 years of Census date), and CanCHEC cycle (i.e., year of cohort entry). We also accounted for rurality, in which participants were classified as living in an urban area if they resided in a Census metropolitan area (CMA) or a moderate-strong metropolitan influence zone (MIZ), and rural if they did not.

### Statistical analysis

We completed a descriptive analysis to determine the total and proportion of participants belonging to each neighbourhood income quintile and to the low-income (LICO) and non-low-income groups by each of the covariates. In doing so, we can determine the distribution of low-income individuals in each neighbourhood income quintile. To examine the association between income and hospitalization, we used Poisson regression models with a person-time offset to find the incidence rate ratio (IRR) of pedestrian, bicycling, or motor vehicle hospitalizations separately. For each type of hospitalization (pedestrian, bicycling and motor vehicle), we created 4 models (12 models total). Each model was adjusted for the covariates age, sex, racialization, rurality, recent immigration, CanCHEC cycle, commute mode and an offset of person-days. For each type of hospitalization, income was measured in 4 ways: Model 1: individual low-income status (LICO), Model 2: neighbourhood income quintile, Model 3: both individual low-income status and neighbourhood income quintile and Model 4: individual low-income status, neighbourhood income quintile and the interaction of the two. Model 4 was used to predict and plot the number of hospitalizations per 10,000 person-days to compare the predicted risk for low-income and non-low-income individuals in each neighbourhood-income quintile.

### Ethics

In accordance with Article 2.4 of the Tri-Council Policy Statement (TCPS), research using entirely anonymized secondary data is exempt from Research Ethics Board (REB) review. Nonetheless, our study protocol was submitted to and reviewed by the Toronto Metropolitan University REB to ensure compliance with TCPS guidelines [34]. As we are using entirely anonymized secondary data a declaration for human ethics and consent to participate as well as consent to publish are not applicable.

## Results

### Descriptive statistics

The cohort consisted of ~ 6,557,000 participants over the 2006, 2011 and 2016 Census cohorts. Of these participants, ~ 65,000 died during the study period, ~ 1,000 had a pedestrian-related hospitalization, ~ 4,000 had a bicycling-related hospitalization, and ~ 7,000 had a motor vehicle occupant/driver-related hospitalization. In total 71.4% of pedestrian hospitalizations were indicated by ICD coding as “in traffic” and 28.6% were indicated as “not in traffic” or “unspecified”. Comparatively more bicycling-related injuries were coded “not in traffic” or “unspecified” (55.6%) while most motor vehicle injuries were considered “in traffic” (82.2%). The largest proportion of non-low-income participants (23.5%) were between the ages of 45–54, while for low-income participants, the largest proportion (25.8%) were aged 15–24. Sex was split fairly evenly for both low and non-low-income participants. 19.7% of non-low-income participants were visible minorities, and 3.3% were Indigenous. While 39.2% of low-income participants were visible minorities and 5.2% were Indigenous. Additionally, 3.1% of non-low-income participants, compared to 13.1% of low-income participants, were recent immigrants. Most participants resided in an urban area and most commuted by car, truck, or van, although a significant number of low-income participants (26.8%) commuted by an “other mode” (Table 1).

**Table 1:** Cohort characteristics by low-income status (LICO) and neighbourhood income quintile in Canadian adults

*N* = 6,557,000		LICO		Neighbourhood Income Quintile				
		Non-low-income (*n* = 6,228,000)	Low-income (*n* = 329,000)	Q1 (lowest) (*n* = 1,044,000)	Q2 (*n* = 1,258,000)	Q3 (*n* = 1,365,000)	Q4 (*n* = 1,450,000)	Q5 (highest) (*n* = 1,440,000)
		(count and %)	(count and %)	(count and %)	(count and %)	(count and %)	(count and %)	(count and %)
**LICO**	**Non-low-income**	6,228,000 (100)	0 (0)	937,000 (89.8)	1,184,000 (94.1)	1,307,000 (95.8)	1,401,000 (96.6)	1,399,000 (97.2)
	**Low-income**	0 (0)	329,000 (100)	108,000 (10.3)	74,000 (5.9)	57,000 (4.2)	49,000 (3.4)	41,000 (2.9)
**Neighbourhood Income Quintile**	**Q1 (lowest)**	937,000 (15.0)	108,000 (32.8)	1,044,000 (100)	0 (0)	0 (0)	0 (0)	0 (0)
	**Q2**	1,184,000 (19.0)	74,000 (22.5)	0 (0)	1,258,000 (100)	0 (0)	0 (0)	0 (0)
	**Q3**	1,307,000 (21.0)	57,000 (17.3)	0 (0)	0 (0)	1,365,000 (100)	0 (0)	0 (0)
	**Q4**	1,401,000 (22.5)	49,000 (14.9)	0 (0)	0 (0)	0 (0)	1,450,000 (100)	0 (0)
	**Q5 (highest)**	1,399,000 (22.5)	41,000 (12.5)	0 (0)	0 (0)	0 (0)	0 (0)	1,440,000 (100)
**Age Group**	**15–24**	925,000 (14.9)	85,000 (25.8)	161,000 (15.4)	188,000 (14.9)	205,000 (15.0)	221,000 (15.2)	235,000 (16.3)
	**25–34**	1,222,000 (19.6)	80,000 (24.3)	240,000 (23.0)	271,000 (21.5)	280,000 (20.5)	279,000 (19.2)	231,000 (16.0)
	**35–44**	1,330,000 (21.4)	67,000 (20.4)	219,000 (21.0)	263,000 (20.9)	295,000 (21.6)	321,000 (22.1)	299,000 (20.8)
	**45–54**	1,466,000 (23.5)	56,000 (17.0)	222,000 (21.3)	281,000 (22.3)	314,000 (23.0)	344,000 (23.7)	360,000 (25.0)
	**55–64**	1,011,000 (16.2)	36,000 (10.9)	157,000 (15.0)	200,000 (15.9)	214,000 (15.7)	228,000 (15.7)	247,000 (17.2)
	**65+**	274,000 (4.4)	6,000 (1.8)	44,000 (4.2)	54,000 (4.3)	56,000 (4.1)	58,000 (4.0)	68,000 (4.7)
**Sex**	**Female**	3,034,000 (48.7)	168,000 (51.1)	515,000 (49.3)	619,000 (49.2)	667,000 (48.9)	707,000 (48.8)	694,000 (48.2)
	**Male**	3,194,000 (51.3)	161,000 (48.9)	529,000 (50.7)	639,000 (50.8)	698,000 (51.1)	743,000 (51.2)	746,000 (51.8)
**Racialization**	**White**	4,799,000 (77.1)	184,000 (55.9)	677,000 (64.8)	903,000 (71.8)	1,030,000 (75.5)	1,158,000 (79.9)	1,215,000 (84.4)
	**Visible minority**	1,225,000 (19.7)	129,000 (39.2)	316,000 (30.3)	309,000 (24.6)	291,000 (21.3)	251,000 (17.3)	187,000 (13.0)
	**Indigenous**	205,000 (3.3)	17,000 (5.2)	52,000 (5.0)	46,000 (3.7)	44,000 (3.2)	42,000 (2.9)	37,000 (2.6)
**Recent Immigration**	**No**	6,036,000 (96.9)	286,000 (86.9)	969,000 (92.8)	1,203,000 (95.6)	1,321,000 (96.8)	1,414,000 (97.5)	1,415,000 (98.3)
	**Yes**	193,000 (3.1)	43,000 (13.1)	75,000 (7.2)	55,000 (4.4)	43,000 (3.2)	36,000 (2.5)	25,000 (1.7)
**Commute Mode**	**Car**,** truck**,** van**	5,071,000 (81.4)	193,000 (58.7)	729,000 (69.8)	979,000 (77.8)	1,120,000 (82.1)	1,223,000 (84.3)	1,213,000 (84.2)
	**Bicyclist**	77,000 (1.2)	8,000 (2.4)	16,000 (1.5)	17,000 (1.4)	17,000 (1.2)	16,000 (1.1)	19,000 (1.3)
	**Pedestrian**	332,000 (5.3)	40,000 (12.2)	91,000 (8.7)	79,000 (6.3)	69,000 (5.1)	64,000 (4.4)	68,000 (4.7)
	**Other**	749,000 (12.0)	88,000 (26.7)	208,000 (19.9)	183,000 (14.5)	159,000 (11.6)	147,000 (10.1)	139,000 (9.7)
**Rurality**	**Urban**	5,222,000 (83.8)	296,000 (90.0)	883,000 (84.6)	1,056,000 (83.9)	1,150,000 (84.2)	1,227,000 (84.6)	1,203,000 (83.5)
	**Rural**	1,006,000 (16.2)	33,000 (10.0)	162,000 (15.5)	202,000 (16.1)	215,000 (15.8)	223,000 (15.4)	237,000 (16.5)
**CanCHEC Cycle**	**2006**	1,644,000 (26.4)	100,000 (30.4)	279,000 (26.7)	336,000 (26.7)	364,000 (26.7)	387,000 (26.7)	379,000 (26.3)
	**2011**	1,635,000 (26.3)	71,000 (21.6)	249,000 (23.9)	316,000 (25.1)	356,000 (26.1)	397,000 (27.4)	388,000 (26.9)
	**2016**	2,949,000 (47.4)	157,000 (47.7)	516,000 (49.4)	606,000 (48.2)	645,000 (47.3)	666,000 (45.9)	673,000 (46.7)

LICO = low-income cut-off, CanCHEC = Canadian Census Health and Environment Cohorts

### Distribution of low-income individuals by neighbourhood income quintile

Approximately 5.0% of analytic cohort members were classified as low-income based on individual income (Table 1). While 32.8% of low-income cohort members resided in the lowest-income neighbourhoods, a substantial number were found in all quintiles, with 12.5% of low-income individuals residing in the highest-income quintile neighbourhoods (Table 1).

### Associations between income and transportation injury

The Poisson model results are shown in Table 2. For pedestrian injury and motor vehicle injury outcomes, both individual and neighbourhood low-income showed relationships when modelled separately. Individual low income (Model 1) was associated with pedestrian hospitalization (IRR = 1.93, 95% CI (1.62, 2.29)) and motor vehicle hospitalization (IRR = 1.18, 95% CI (1.06, 1.31)); for neighbourhood income (Model 2), the lowest quintile had an estimated IRR = 1.80, 95% CI (1.53, 2.13), compared to Q5 (highest neighbourhood income quintile) for pedestrian injury, and IRR = 1.33, 95% CI (1.23, 1.44) for motor vehicle injury. For injuries by both these transportation modes, there appears to be a gradient, where the lower the neighbourhood income quintile, the higher the risk. Including both individual income and neighbourhood income in the same model (Table 2, Model 3) yielded similar patterns of association.

**Table 2:** Incidence rate ratio for pedestrian, bicycling, and motor vehicle hospitalizations in Canadian adults

Pedestrian Hospitalization									
		Model 1^a^		Model 2^b^		Model 3^c^		Model 4^d^	
		IRR	95% CI for IRR	IRR	95% CI for IRR	IRR	95% CI for IRR	IRR	95% CI for IRR
**LICO**	**Non-low-income**	*ref*				*ref*		*ref*	
	**Low-income**	**1.93**	**(1.62**,** 2.29)**			**1.77**	**(1.49**,** 2.11)**	**3.37**	**(2.22**,** 5.11)**
**Neighbourhood Income Quintile**	**Q5 (highest)**			*ref*		*ref*		*ref*	
	**Q4**			1.01	(0.84, 1.20)	1.00	(0.84, 1.20)	1.06	(0.88, 1.28)
	**Q3**			1.05	(0.88, 1.25)	1.04	(0.87, 1.24)	1.13	(0.94, 1.36)
	**Q2**			**1.35**	**(1.14**,** 1.60)**	**1.33**	**(1.12**,** 1.57)**	**1.41**	**(1.18**,** 1.68)**
	**Q1 (lowest)**			**1.80**	**(1.53**,** 2.13)**	**1.72**	**(1.46**,** 2.03)**	**1.80**	**(1.51**,** 2.14)**
LICO-Neighbourhood Income Quintile Interaction*	**Low-income: Q5**							*ref*	
	**Low-income: Q4**							*0.46*	*(0.23*,* 0.90)*
	**Low-income: Q3**							*0.30*	*(0.15*,* 0.62)*
	**Low-income: Q2**							*0.47*	*(0.27*,* 0.81)*
	**Low-income: Q1**							*0.55*	*(0.34*,* 0.89)*
**Bicycle Hospitalization**									
		**Model 1** ^a^		**Model 2** ^b^		**Model 3** ^c^		**Model 4** ^d^	
		**IRR**	**95% CI for IRR**	**IRR**	**95% CI for IRR**	**IRR**	**95% CI for IRR**	**IRR**	**95% CI for IRR**
**LICO**	**Non-low-income**	*ref*				*ref*		*ref*	
	**Low-income**	**1.16**	**(1.01**,** 1.34)**			**1.20**	**(1.04**,** 1.39)**	0.84	(0.57, 1.24)
**Neighbourhood Income Quintile**	**Q5 (highest)**			*ref*		*ref*		*ref*	
	**Q4**			*0.78*	*(0.71*,* 0.86)*	*0.78*	*(0.71*,* 0.86)*	*0.77*	*(0.70*,* 0.85)*
	**Q3**			*0.71*	*(0.65*,* 0.79)*	*0.71*	*(0.65*,* 0.78)*	*0.71*	*(0.64*,* 0.78)*
	**Q2**			*0.69*	*(0.63*,* 0.77)*	*0.69*	*(0.62*,* 0.76)*	*0.67*	*(0.60*,* 0.74)*
	**Q1 (lowest)**			*0.74*	*(0.67*,* 0.82)*	*0.73*	*(0.66*,* 0.81)*	*0.73*	*(0.65*,* 0.81)*
LICO-Neighbourhood Income Quintile Interaction*	**Low-income: Q5**							*ref*	
	**Low-income: Q4**							1.47	(0.87, 2.49)
	**Low-income: Q3**							1.32	(0.78, 2.24)
	**Low-income: Q2**							**1.93**	**(1.19**,** 3.11)**
	**Low-income: Q1**							1.42	(0.89, 2.27)
**Motor Vehicle Hospitalization**									
		**Model 1** ^a^		**Model 2** ^b^		**Model 3** ^c^		**Model 4** ^d^	
		**IRR**	**95% CI for IRR**	**IRR**	**95% CI for IRR**	**IRR**	**95% CI for IRR**	**IRR**	**95% CI for IRR**
**LICO**	**Non-low-income**	*ref*				*ref*		*ref*	
	**Low-income**	**1.18**	**(1.06**,** 1.31)**			**1.15**	**(1.03**,** 1.27)**	1.28	(0.97, 1.70)
**Neighbourhood Income Quintile**	**Q5 (highest)**			*ref*		*ref*		*ref*	
	**Q4**			**1.17**	**(1.09**,** 1.26)**	**1.16**	**(1.08**,** 1.25)**	**1.16**	**(1.08**,** 1.25)**
	**Q3**			**1.23**	**(1.15**,** 1.32)**	**1.23**	**(1.14**,** 1.32)**	**1.23**	**(1.14**,** 1.32)**
	**Q2**			**1.23**	**(1.14**,** 1.32)**	**1.22**	**(1.13**,** 1.31)**	**1.23**	**(1.14**,** 1.32)**
	**Q1 (lowest)**			**1.33**	**(1.23**,** 1.44)**	**1.31**	**(1.21**,** 1.41)**	**1.33**	**(1.22**,** 1.44)**
LICO-Neighbourhood Income Quintile Interaction*	**Low-income: Q5**							*ref*	
	**Low-income: Q4**							1.08	(0.75, 1.56)
	**Low-income: Q3**							0.89	(0.61, 1.28)
	**Low-income: Q2**							0.83	(0.58, 1.18)
	**Low-income: Q1**							0.82	(0.58, 1.15)

LICO = low-income cut-off, IRR = incidence rate ratio, CI = confidence interval. Bolded IRR = statistically significant elevated, Italicized IRR = statistically significant lowered

*The interaction must be interpreted with caution; the values are used as an adjustment factor to determine the IRR for low-income individuals compared to non-low-income individuals within the same neighbourhood income quintile

^a^ **Model 1** adjusts for individual low-income status (LICO) and the covariates: age, sex, commute mode, racialization, recent immigration, rurality and CanCHEC cycle

^b^ **Model 2** adjusts for neighbourhood income quintile and the covariates: age, sex, commute mode, racialization, recent immigration, rurality and CanCHEC cycle

^c^ **Model 3** adjusts for individual low-income status (LICO), neighbourhood income quintile and the covariates: age, sex, commute mode, racialization, recent immigration, rurality and CanCHEC cycle

^d^ **Model 4** adjusts for individual low-income status (LICO), neighbourhood income quintile, their interaction and the covariates: age, sex, commute mode, racialization, recent immigration, rurality and CanCHEC cycle

Like the other transportation modes, for bicycling injury individual income elevated risk when measured alone (IRR = 1.16, 95% CI (1.01, 1.34)) and remained elevated when neighbourhood income was added to the model (Model 3). The relationship between cycling and neighbourhood income (Model 2) was different than the other transportation modes and it suggested decreasing risk at lower neighbourhood income quintiles. The pattern observed for neighbourhood income was robust to the inclusion of individual income in the same model (Table 2, Model 3).

### Interaction between individual income and neighbourhood income quintile

**Fig. 1:**
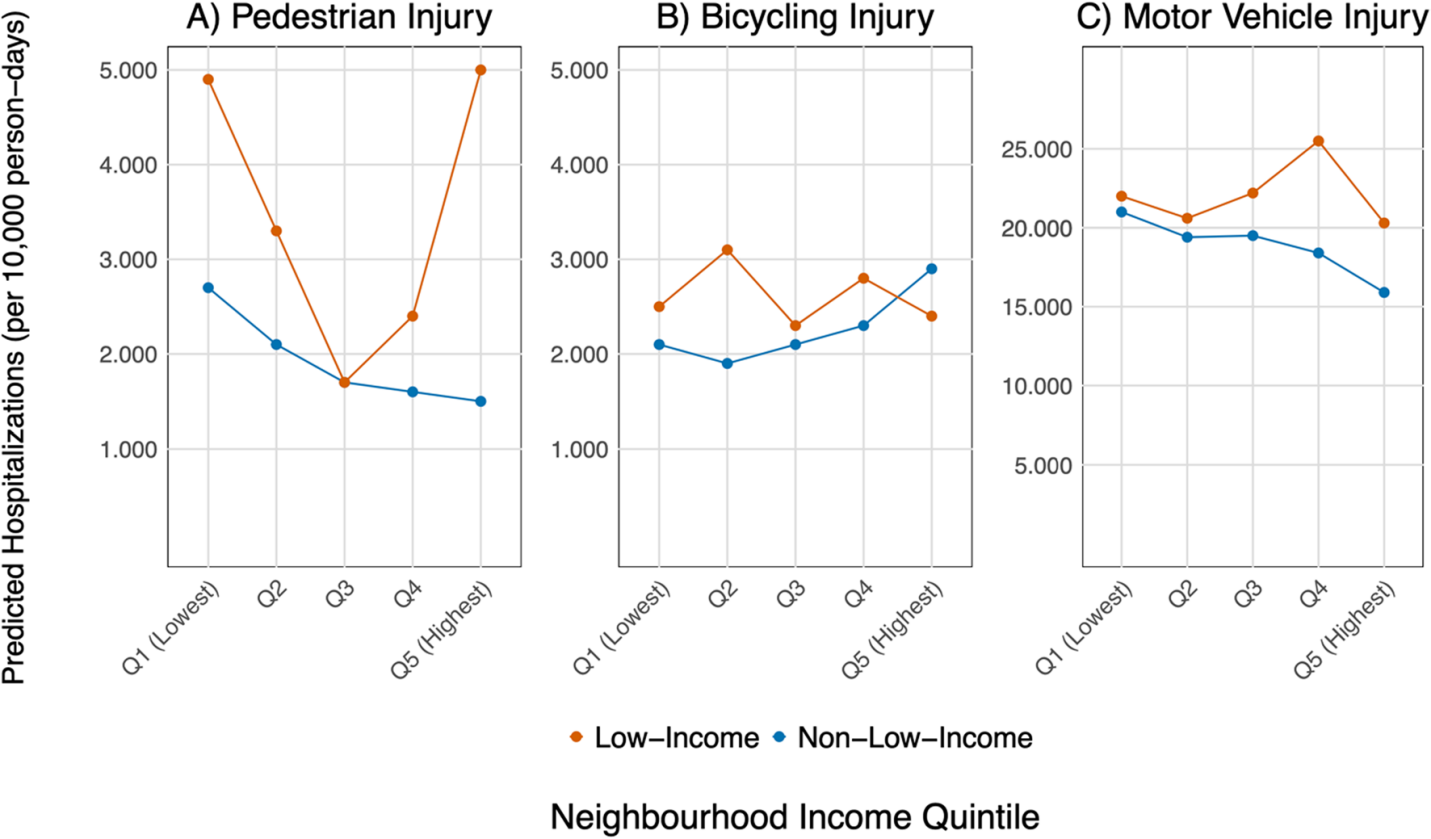
Predicted number of hospitalizations per 10,000 person-days from the Poisson regression model (Model 4), using individual low-income status, neighbourhood income quintile, and their interaction, adjusted for sex, age, racialization, recent immigration, rurality, CanCHEC cycle, commute mode and an offset of 1,789 days. Panels are separated by injury type: (**A**) Pedestrian injury, (**B**) Bicycling injury and (**C**) Motor vehicle injury

The coefficients for Model 4 are shown in Table 2, and Fig. 1 depicts the interaction between individual income and neighbourhood income quintiles graphically. Rates of injury and the relationship to neighbourhood income for lower-income individuals show different patterns than those predicted for non-low-income individuals. In non-low-income individuals (above LICO), predicted rates of injury show monotonic relationships with neighbourhood income quintile, with progressively lower predicted rates with increasing neighbourhood income quintile for pedestrian and motor vehicle injuries and progressively increasing predicted rates for bicycling injury. However, among low-income individuals, the association between predicted injury rates and neighbourhood income is non-monotonic, with intermediate neighbourhood income quintiles showing lower predicted rates for pedestrian injury and mixed results for bicycling and motor vehicle injury. Furthermore, as depicted in Fig. 1, low-income individuals in the second-highest-income neighbourhoods (Q4) have the greatest inequity in predicted motor vehicle injuries. While low-income individuals in the highest-income neighbourhood (Q5) experienced the greatest inequity for pedestrian-related injuries, with low-income individuals at more than three times the rate of predicted pedestrian injuries in comparison to non-low-income individuals in the same neighbourhood quintile.

## Discussion

This study analyzed associations between individual low-income status and neighbourhood income quintile on transportation injury in Canadian adults. We found individual low-income status and neighbourhood income show independent and joint associations with pedestrian, bicycle, and motor vehicle hospitalizations. Our results suggest that people with low income are at greater risk of pedestrian, bicyclist and motor vehicle injury, no matter where they live, excluding bicycling injuries in high-income neighbourhoods. The greatest inequity in the impact of individual income is seen for pedestrian injuries in the wealthiest neighbourhoods, where low-income individuals experience more than triple the incidence of injuries in comparison to their non-low-income counterparts. We do not assume here that income (by either construct) directly causes injury; below we consider possible interpretations of the income relationships shown and the difference between individually and ecologically assessed income measures.

### Heterogeneity within neighbourhood income quintiles

It is rare for individual income data to be available in administrative injury records in Canada. Therefore, many studies have relied on the location of injury (or location of residence) and the associated area-level estimates of income [35]. Similar to the findings by Hanley and Morgan [36], people with low incomes reside across every neighbourhood income quintile, indicating that the assumption that average neighbourhood income can be used as a proxy for individual income does not hold. Using only area-level measures of income to predict risk may inaccurately categorize the individuals in that area. We cannot assume, for example, that all people who reside in wealthy neighbourhoods have low risk, as there are important risk factors independent of that area-level measure.

Previous studies have indicated that those who live in low-income areas are at greater risk of transportation injuries [7]. This increased risk is suggested to be the result of unsafe infrastructure designs and transportation planning specific to lower-income areas. For example, traffic calming measures (i.e. speed humps) have been shown to reduce pedestrian injuries [37], and there are fewer of these measures in lower-income areas [9]. Furthermore, low-income neighbourhoods have increased traffic volume at intersections, increasing risk of road-user injury [7]. However, in analyzing these injury outcomes at the individual level, we found neighbourhood income quintile conveyed different information on risk than individual income.

### The impact of individual income on transportation injury risk

There are fewer studies of injury risk by individual income status (independent of neighbourhood income) because the data are rarely available, however, some studies have shown that individual measures tend to yield stronger inequity estimates [5]. In our study, individual low-income appeared to exert an independent influence on risk for all 3 outcomes. This suggests that for people with low income, there is a different underlying risk than for their non-low-income counterparts, regardless of their neighbourhood income. Our interaction analysis showed that there are large inequities in injury occurring in the highest-income neighbourhoods, indicating the health benefits of living in a high-income area are less effective for those who are individually low-income, a finding mirrored by another recent Canadian cohort study on avoidable hospitalizations [38]. One potential explanation for this increased risk among low-income pedestrians residing in high-income areas may be related to increased exposure to risk (e.g., more walking), where low-income individuals may be less likely to own a car and more likely to walk [39]. 

### How individual income may impact transportation injury risk

Next, we broadly consider how individual income may impact risk. Low income is a risk factor for poorer overall health, including previous injuries and comorbidities, which could increase the risk of future injuries as well as severity of injuries [2, 10]. 

Fatigue is another large issue for all road users, especially those in motor vehicles [13]. Low-income individuals are more likely to experience fatigue due to long or irregular working hours, shift work, and lack of access to fatigue management resources [14]. Shift work may also result in commuting during the night, which is associated with fatal traffic injuries [16]. Additionally, low-income individuals are more likely to have extreme commute times, exceeding 60 min, increasing exposure [17]. 

The impact of individual income on mental health may also be a factor. Low-income individuals are more likely to experience chronic stress due to a variety of factors, which is a predictor of traffic injury rates [11, 12]. 

Additionally, low-income individuals are also more likely to have older, less safe models of cars, while more expensive models are larger and heavier, increasing risk of fatal crashes for smaller models [15, 40]. 

### How bicycling injury risk differs from pedestrian and motor vehicle injury risk

The results for bicycling showed a different relationship in comparison to the other outcomes. We found the highest risk for low-income individuals to be in the second lowest income quintile, while non-low-income individuals had the highest risk in the wealthiest neighbourhoods. Low-income individuals were generally at higher risk than non-low-income individuals in all-income neighbourhoods except the wealthiest. Cycling requires access to a personal bike or bike share program and higher-income areas tend to have greater access to bike share programs in Canada [8]. This may help explain the increased risk for all people who live in high-income areas, as there would be greater access. For low-income individuals, helmet use may play a role. Helmet use has been found to be positively associated with income in both recreational and transportation cycling [41]. 

### Strengths and limitations

This study has many strengths, including access to both individual and area-level measures of income. Another strength is the use of a standardized indicator of low income (LICO) designed to account for the differential cost of living in different regions of Canada. Additionally, by linking Census variables to health outcomes, studies using CanCHECs can include rich sociodemographic covariates. Uniquely, we were also able to adjust for an indicator of the use of individual modes of transportation by including commute mode as a covariate [32]. 

This analysis has limitations. First, those with missing or unmatched postal codes, as well as those with a missing individual income status (those in the territories and on reserves), were excluded from these analyses. To compare how results may have changed without the removal of these groups, we completed a sensitivity analysis with an additional “missing” category for both individual low-income status and neighbourhood income quintile. The reported relationships were unchanged by this addition, with “missing” income showing similar risk profiles to lower income categories.

We have limited information about the circumstances under which the included injuries occurred. We used the broadest categories of ICD coding to indicate relevant injuries, with some pedestrian, cyclist, and motor vehicle injuries categorized as “not in traffic”. Among pedestrian injuries detected by ICD coding in health databases, almost all reflect collisions with motor vehicles, whereas bicycle injuries may include crashes on or off-road [42]. Collisions with motor vehicles occurring “not in traffic” and single bicycle crashes occurring on-road “in traffic” make the “in traffic” indicator complex to interpret. Given the lack of detailed information on how coding is used to record injuries across injury types, we used an inclusive definition of these injuries to ensure relevant injuries were captured that often do not appear in police-reported collision records [43]. 

Residential postal codes present in the data sets may not reflect the locations where the collisions occurred. Therefore, the neighbourhood income level may not represent the collision environment as we have assumed in this analysis. Similarly, if a participant used a mailing address other than their residence on their tax forms, then the postal code may not reflect where they live, although this would represent a small subset of the sample [18, 44]. Studies relying police collision reports have reliable and consistent detail on the location of injury; however, the limited police attendance at crashes mean these reports miss many transportation injuries [43]. 

A particular limitation of reported commute mode is that it may change depending on the time of year, with the Census conducted in May, and data collected at that time. A limitation of the use of multiple Census cohorts is the potential to include repeat participants who were captured in multiple Census cycles.

Finally, the study findings are generalizable only to the specific population under study: Canadian commuters. Our hospitalization analyses are also not generalizable to Québec residents due to the absence of Québec reporting in national hospitalization admissions data [45]. Ongoing efforts to harmonize administrative health databases across Canadian provinces will further improve the depth and generalizability of linkage studies.

## Conclusion

The findings of this study highlight the unique impact individual income may have on transportation injury risk, warranting the need for future studies to include both individual and area-level measures of income. Further study into the mechanisms by which individual income can impact risk is also needed. Additionally, continued linkage between all types of transportation collision records, including police and hospitalization records, and individual-level data like the Census, can help to better understand the causes of transportation injury.

## Supplementary Information

Supplementary Material 1

## Data Availability

Approved researchers can access the data in the Statistics Canada Research Data Centre. Information about the RDC is available at https://www.statcan.gc.ca/en/microdata/data-centres.

## Abbreviations

CanCHECs: Canadian Census Health and Environment Cohorts
NHS: National Household Survey
DAD: Discharge Abstract Database
CVSD: Canadian Vital Statistics Death Database
PCCF+: Postal Code Conversion File Plus
LICO: Low-income cut-off
DA: Dissemination area
ICD-10: International Statistical Classification of Diseases and Related Health Problems, 10th Revision
CMA: Census metropolitan area
MIZ: Metropolitan influence zone
IRR: Incidence rate ratio
CI: Confidence interval
