# Supplementary material for: Measuring inequities in transportation injuries in a Canadian commuter cohort: impacts of individual versus neighbourhood income
Source: Inj Epidemiol. 2025 Oct 1;12:64. doi: 10.1186/s40621-025-00615-8 (PMC12487150; doi:10.1186/s40621-025-00615-8)
Supplement: Supplementary file 1 — Supplementary Material 1 [file 40621_2025_615_MOESM1_ESM.docx]

**Appendix A**

**Table 1A:** ICD-10 codes for bicycling, pedestrian and motor vehicle occupant/driver-related injuries.

| Outcome type | ICD-10 |
| --- | --- |
| **Bicycling-related** | V100; V101; V102; V103; V104; V105; V109; V110; V111; V112; V113; V114; V115; V119; V120; V121; V122; V123; V124; V125; V129; V130; V131; V132; V133; V134; V135; V139; V140; V141; V142; V143; V144; V145; V149; V150; V151; V152; V153; V154; V155; V159; V160; V161; V162; V163; V164; V165; V169; V170; V171; V172; V173; V174; V175; V179; V180; V181; V182; V183; V184; V185; V189; V190; V191; V192; V193; V194; V195; V196; V198; V199 |
| **Pedestrian-related** | V010; V011; V019; V020; V021; V029; V030; V031; V039; V040; V041; V049; V050; V051; V059; V060; V061; V069; V090; V091; V092; V093; V099 |
| **Motor vehicle occupant/driver-related** | V400; V401; V403; V404; V405; V406; V409; V410; V411; V413; V414; V415; V416; V419; V420; V421; V423; V424; V425; V426; V429; V430; V431; V433; V434; V435; V436; V439; V440; V441; V443; V444; V445; V446; V449; V450; V451; V453; V454; V455; V456; V459; V460; V461; V463; V464; V465; V466; V469; V470; V471; V473; V474; V475; V476; V479; V480; V481; V483; V484; V485; V486; V489; V490; V491; V492; V493; V494; V495; V496; V498; V499; V500; V501; V503; V504; V505; V506; V509; V510; V511; V513; V514; V515; V516; V519; V520; V521; V523; V524; V525; V526; V529; V530; V531; V533; V534; V535; V536; V539; V540; V541; V543; V544; V545; V546; V549; V550; V551; V553; V554; V555; V556; V559; V560; V561; V563; V564; V565; V566; V569; V570; V571; V573; V574; V575; V576; V579; V580; V581; V583; V584; V585; V586; V589; V590; V591; V592; V593; V594; V595; V596; V598; V599; V600; V601; V603; V604; V605; V606; V609; V610; V611; V613; V614; V615; V616; V619; V620; V621; V623; V624; V625; V626; V629; V630; V631; V633; V634; V635; V636; V639; V640; V641; V643; V644; V645; V646; V649; V650; V651; V653; V654; V655; V656; V659; V660; V661; V663; V664; V665; V666; V669; V670; V671; V673; V674; V675; V676; V679; V680; V681; V683; V684; V685; V686; V689; V690; V691; V692; V693; V694; V695; V696; V698; V699 |

*Adapted from *Branion-Calles et al.*^28^
